# Supplementary material for: Genomic Characterization of Multiple Antibiotic-Resistant Enterococcus in Farm Animals in Ningxia Province, China
Source: Antibiotics (Basel). 2025 Nov 10;14(11):1137. doi: 10.3390/antibiotics14111137 (PMC12649137; doi:10.3390/antibiotics14111137)
Supplement: Supplementary file 1 [file antibiotics-14-01137-s001.zip › antibiotics-3885085-supplementary.pdf]

Table S1 Isolation of *Enterococcus* in Ningxia Province, China.

Years	Animal species	Gram-positive bacteria (G +)		
		Enterococcus.sp		
		Enterococcus faecalis	*Enterococcus faecium*	Other *Enterococcus*
2019	cattle	3	13	43
2020		2	11	74
2021		8	16	120
2022		6	9	120
2023		7	11	163
2023	Beef cattle	7	4	31
2020	sheep	17	3	11
2021		0	0	33
2022		5	16	42
2023		2	6	10
2019	swine	0	0	6
2020		13	7	9
2021		39	0	1

2023		38	4	9
2019	chicken	16	1	2
2020		169	52	35
2021		150	36	31
2022		24	16	4
2023		34	9	10
total		540	214	754

Table 2 Antibiotics resistance spectrum of *Enterococcus.*

strain	Spectrum of drug resistance
NXC1	--ERY---SF--DOX-TIA-TIL-
NXC2	--ERY-ENR--SF--DOX-TIA-TIL-
NXC3	--ERY---SF--DOX-TIA-TIL-
NXC5	--ERY---SF---TIA-TIL-
NXC7	--ERY-ENR--SF---TIA-TIL-
NXC8	--ERY-ENR--SF--DOX-TIA-TIL-
NXC9	--ERY-ENR--SF---TIA-TIL-
NXC10	--ERY-ENR--SF--DOX-TIA-TIL-
NXC11	--ERY-ENR--SF---TIA-TIL-LZD
NXC12	--ERY---SF--DOX-TIA-TIL-
NXC13	PEN--ERY---SF--DOX-TIA-TIL-
NXC14	--ERY---SF--DOX-TIA-TIL-
NXC15	--ERY---SF---TIA--
NXC16	--ERY---SF--DOX-TIA-TIL-
NXC17	--ERY-ENR--SF--DOX-TIA-TIL-
NXC18	--ERY---SF--DOX-TIA-TIL-

NXC19	--ERY-ENR--SF---TIA-TIL-
NXC20	--ERY---SF--DOX-TIA-TIL-
NXC21	--ERY---SF---TIA-TIL-LZD
NXC22	--ERY---SF---TIA-TIL-
NXC23	--ERY-ENR--SF---TIA-TIL-
NXC27	--ERY-ENR--SF---TIA-TIL-
NXC30	PEN--ERY---SF--DOX-TIA-TIL-
NXC32	--ERY-ENR--SF---TIA-TIL-
NXC33	--ERY-ENR--SF--DOX-TIA-TIL-
NXC34	--ERY---SF---TIA-TIL-
NXC35	--ERY---SF--DOX-TIA-TIL-
NXC36	--ERY---SF--DOX-TIA-TIL-
NXC37	--ERY-ENR--SF--DOX-TIA-TIL-
NXC38	--ERY---SF---TIA-TIL-
NXC39	--ERY---SF--DOX-TIA-TIL-LZD
NXC40	--ERY-ENR--SF---TIA-TIL-
NXC41	-----SF---TIA-TIL-
NXC42	--ERY---SF-VAN--TIA-TIL-LZD

NXC43	--ERY-ENR--SF----TIL-
NXC44	--ERY---SF---TIA--
NXC45	--ERY---SF---TIA-TIL-
NXC46	-----SF---TIA-TIL-
NXC47	--ERY---SF---TIA-TIL-
NXC48	---ENR--SF---TIA-TIL-
NXC49	---ENR--SF---TIA-TIL-
NXC50	--ERY---SF---TIA-TIL-
NXC51	---ENR--SF---TIA-TIL-
NXC52	--ERY---SF---TIA-TIL-
NXC53	-A/C-ERY---SF---TIA--
NXC54	--ERY---SF---TIA-TIL-
NXC55	---ENR--SF--DOX-TIA-TIL-
NXC56	--ERY-ENR--SF---TIA-TIL-
NXC57	--ERY---SF-VAN--TIA-TIL-LZD
NXC58	--ERY-ENR--SF--DOX-TIA-TIL-
NXC59	--ERY-ENR--SF--DOX-TIA-TIL-
NXC60	--ERY---SF---TIA--

NXC61	--ERY---SF---TIA--
NXC62	-----SF---TIA-TIL-
NXC63	-----SF---TIA-TIL-
NXC64	--ERY---SF---TIA-TIL-
NXC65	--ERY---SF--DOX-TIA-TIL-
NXC66	--ERY---SF-VAN--TIA-TIL-LZD
NXC67	--ERY---SF--DOX-TIA-TIL-LZD
NXC71	PEN---ENR--SF--DOX-TIA-TIL-
NXC72	-----SF---TIA-TIL-
NXC73	---ENR-----TIA-TIL-
NXC74	-----SF---TIA-TIL-
NXC75	-----SF---TIA-TIL-
NXC76	--ERY---SF---TIA--
NXC77	-----SF---TIA-TIL-
NXC78	-----SF---TIA-TIL-
NXC81	--ERY---SF--DOX-TIA-TIL-LZD
NXC84	--ERY---SF--DOX-TIA-TIL-
NXC85	--ERY-ENR--SF--DOX-TIA-TIL-

NXC86	--ERY-ENR--SF--DOX-TIA-TIL-LZD
NXC87	--ERY-ENR--SF--DOX-TIA-TIL-LZD
NXC88	--ERY-ENR--SF---TIA-TIL-
NXC89	--ERY---SF--DOX-TIA-TIL-LZD
NXC90	--ERY-ENR--SF--DOX-TIA-TIL-LZD
NXC91	--ERY---SF--DOX-TIA-TIL-LZD
NXC92	PEN-A/C-ERY-ENR--SF---TIA-TIL-LZD
NXC95	--ERY-ENR--SF--DOX-TIA-TIL-LZD
NXC99	--ERY-ENR--SF---TIA-TIL-LZD
NXC101	--ERY-ENR--SF--DOX-TIA-TIL-
NXC102	--ERY---SF--DOX-TIA-TIL-
NXGC33	--ERY---SF--DOX-TIA-TIL-
NXGC34	-----SF---TIA--
NXGC35	--ERY---SF--DOX-TIA-TIL-
NXGC36	--ERY---SF--DOX-TIA-TIL-
NXGC37	--ERY-ENR--SF--DOX-TIA-TIL-
NXGC38	--ERY---SF---TIA-TIL-LZD

NXGC39	--ERY-ENR--SF---TIA-TIL-
NXGC40	--ERY---SF---TIA--

Table 3 *Enterococcus* virulence gene antibiotics resistance genes statistics.

strain	antibiotics resistance genes	virulence genes
NXC 101	aac(6')-aph(2''),aph(3')-III,cat,dfrG,erm(B),lnu(B),lsa(A),lsa(E),str,tet(L),tet(M)	EF3023,bopD,cpsA,cpsB,ebpA,ebpB,ebpC,efaA,fss1,srtC
NXC 102	ant(6)-Ia,aph(3')-III,cat,dfrG,erm(A),erm(B),fexA,lsa(A),optrA,tet(L),tet(M)	EF0818,EF3023,ace,bopD,cpsA,cpsB,ebpA,ebpB,ebpC,efaA,fsrA,fsrB,fsrC,fss1,gelE,prgB/asc10,sprE,srtC
NXC 10	aac(6')-aph(2''),ant(6)-Ia,erm(B),lnu(B),lsa(A),lsa(E),tet(L),tet(M)	EF0818,asa1,bopD,cpsA,cpsB,cpsC,cpsD,cpsE,cpsG,cpsH,cpsI,cpsJ,cpsK,cylA,cylB,cylI,cylL,cylM,cylR1,cylR2,cylS,ebpA,ebpB,ebpC,efaA,fsrA,fsrB,fsrC,fss1,gelE,sprE,srtC
NXC 11	ant(6)-Ia,ant(9)-Ia,aph(3')-III,cat,dfrG,erm(A),fexA,lsa(A),optrA,tet(L),tet(M)	EF0485,EF3023,bopD,cpsA,cpsB,ebpA,ebpB,ebpC,efaA,fss1,gelE,sprE,srtC
NXC 12	aac(6')-Ii,msr(C),tet(S)	acm

NXC 13	aac(6')-Ii,msr(C)	acm,scm
NXC 14	aac(6')-Ii,msr(C)	scm
NXC 15	aac(6')-Ii,msr(C)	acm,sgrA
NXC 16	dfrG,erm(B),lsa(A),tet(L),tet(M)	EF0149,EF0485,EF0818,bopD,cpsA,cpsB,cpsC,cpsD,cpsE,cpsG,cpsH,cpsI,cpsJ,cpsK,cylI,ebpA,ebpB,ebpC,efaA,fsrA,fsrB,fsrC,fss1,fss2,gelE,sprE,srtC
NXC 17	aac(6')-Ii,ant(6)-Ia,erm(B),lnu(B),lsa(E),msr(C),tet(L),tet(M)	acm
NXC 18	ant(6)-Ia,aph(3')-III,erm(B),lnu(G),lsa(A),tet(L),tet(M)	EF3023,bopD,cpsA,cpsB,cpsC,cpsD,cpsE,cpsF,cpsG,cpsH,cpsI,cpsJ,cpsK,ebpA,ebpB,ebpC,efaA,fsrA,fsrB,fsrC,fss1,gelE,prgB/asc10,sprE,srtC
NXC 19	cat,erm(B),lsa(A),tet(M)	EF3023,bopD,cpsA,cpsB,cpsC,cpsD,cpsE,cpsF,cpsG,cpsH,cpsI,cpsJ,cpsK,cylA,cylB,cylI,cylL,cylM,cylR1,cylR2,cylS,ebpA,ebpB,ebpC,efaA,fsrA,fsrB,fsrC,fss1,fss2,gelE,sprE,srtC

NXC 1	aac(6')-aph(2''),aph(3')-III,dfrG,lnu(B),lsa(A),lsa(E),str,tet(L),tet(M)	EF3023,bopD,cpsA,cpsB,cpsC,cpsD,cpsE,cpsF,cpsG,cpsH,cpsI,cpsJ,cpsK,ebpA,ebpB,ebpC,efaA,fsrA,fsrB,fsrC,fss1,fss2,gelE,sprE,srtC
NXC 20	aac(6')-aph(2''),aph(3')-III,dfrG,lnu(B),lsa(A),lsa(E),str,tet(M)	EF0818,bopD,cpsA,cpsB,ebpA,ebpB,ebpC,efaA,fsrA,fsrB,fsrC,fss1,gelE,prgB/asc10,sprE,srtC
NXC 21	ant(9)-Ia,aph(3')-III,cat,dfrG,erm(A),erm(B),fexA,lsa(A),optrA,tet(L),tet(M)	EF3023,bopD,cpsA,cpsB,cpsC,cpsD,cpsE,cpsF,cpsG,cpsH,cpsI,cpsJ,cpsK,ebpA,ebpB,ebpC,efaA,fsrA,fsrB,fsrC,fss1,gelE,sprE,srtC
NXC 22	aac(6')-aph(2''),aph(3')-III,dfrG,erm(A),fexA,lnu(B),lsa(A),lsa(E),optrA,tet(L),tet(M)	EF0818,ace,bopD,cpsA,cpsB,cpsC,cpsD,cpsE,cpsG,cpsH,cpsI,cpsJ,cpsK,ebpA,ebpB,ebpC,efaA,fsrA,fsrB,fsrC,fss1,fss2,gelE,sprE,srtC
NXC 23	aac(6')-aph(2''),aph(3')-III,cat,dfrG,erm(B),lsa(A),tet(M)	EF0485,EF3023,bopD,cpsA,cpsB,cpsC,cpsD,cpsE,cpsF,cpsG,cpsH,cpsI,cpsJ,cpsK,cylA,cylB,cylI,cylL,cylM,cylR1,cylR2,cylS,ebpA,ebpB,ebpC,efaA,fsrA,fsrB,fss1,gelE,sprE,srtC
NXC 27	lsa(A)	EF0818,EF3023,bopD,cpsA,cpsB,ebpA,ebpB,ebpC,efaA,fsrA,fsrB,fsrC,fss1,gelE,sprE,srtC

NXC 2	aac(6')-aph(2''),aph(3')-III,dfrG,lnu(B),lsa(A),lsa(E),str,tet(L),tet(M)	EF3023,ace,bopD,cpsA,cpsB,cpsC,cpsD,cpsE,cpsF,cpsG,cpsH,cpsI,cpsJ,cpsK,ebpA,ebpB,ebpC,efaA,fsrA,fsrB,fsrC,fss1,fss2,gelE,sprE,srtC
NXC 30	dfrG,erm(B),lsa(A),tet(L),tet(M)	EF0818,bopD,cpsA,cpsB,ebpA,ebpB,ebpC,efaA,fsrA,fsrB,fsrC,fss1,gelE,sprE,srtC
NXC 32	dfrG,erm(B),lsa(A),tet(L),tet(M)	EF0818,bopD,cpsA,cpsB,ebpA,ebpB,ebpC,efaA,fsrA,fsrB,fsrC,fss1,gelE,sprE,srtC
NXC 33	dfrG,erm(B),lsa(A),tet(L),tet(M)	EF0818,bopD,cpsA,cpsB,cpsC,cpsD,cpsE,cpsG,cpsH,cpsI,cpsJ,cpsK,cylI,ebpA,ebpB,ebpC,efaA,fsrA,fsrB,fsrC,fss1,gelE,prgB/asc10,sprE,srtC
NXC 34	erm(B),lsa(A),tet(L),tet(M)	EF0818,EF3023,ace,bopD,cpsA,cpsB,cylI,ebpA,ebpB,ebpC,efaA,fsrA,fsrB,fsrC,fss1,gelE,prgB/asc10,sprE,srtC
NXC 35	cat,lsa(A),tet(L),tet(M)	EF0818,EF3023,ace,bopD,cpsA,cpsB,cylI,ebpA,ebpB,ebpC,efaA,fsrA,fsrB,fsrC,fss1,gelE,prgB/asc10,sprE,srtC
NXC 36	aac(6')-aph(2''),ant(6)-Ia,aph(3')-III,lnu(B),lsa(A),lsa(E),tet(L),tet(M)	EF0818,asa1,bopD,cpsA,cpsB,cpsC,cpsD,cpsE,cpsG,cpsH,cpsI,cpsJ,cpsK,cylA,cylB,cylI,cylL,cylM,cylR1,cylR2,cylS,ebpA,ebpB,ebpC,efaA,fsrA,fsrB,fsrC,fss1,gelE,sprE,srtC

NXC 37	aph(3')-III,dfrG,erm(B),lsa(A),tet(L),tet(M)	EF0818,ace,bopD,cpsA,cpsB,cpsC,cpsD,cpsE,cpsF,cpsG,cpsH,cpsI,cpsJ,cpsK,ebpA,ebpB,ebpC,efaA,fsrA,fsrB,fsrC,fss1,gelE,prgB/asc10,sprE,srtC
NXC 38	aac(6')-aph(2''),ant(6)-Ia,aph(3')-III,lnu(B),lsa(A),lsa(E),optrA,tet(L),tet(M)	EF0818,EF3023,ace,bopD,cpsA,cpsB,ebpA,ebpB,ebpC,efaA,fsrA,fsrB,fsrC,fss1,gelE,prgB/asc10,sprE,srtC
NXC 39	aac(6')-aph(2''),aph(3')-III,erm(A),fexA,lnu(B),lsa(A),lsa(E),optrA,tet(L),tet(M)	EF0818,EF3023,ace,bopD,cpsA,cpsB,ebpA,ebpB,ebpC,efaA,fsrA,fsrB,fsrC,fss1,gelE,sprE,srtC
NXC 3	aac(6')-Ii,msr(C)	acm
NXC 40	aac(6')-aph(2''),aph(3')-III,dfrG,erm(B),lnu(B),lsa(A),lsa(E),tet(L),tet(M)	EF0818,ace,bopD,cpsA,cpsB,cpsC,cpsD,cpsE,cpsF,cpsG,cpsH,cpsI,cpsJ,cpsK,ebpA,ebpB,ebpC,efaA,fsrA,fsrC,fss1,gelE,prgB/asc10,sprE,srtC
NXC 41	lsa(A)	bopD,cpsA,cpsB,cpsC,cpsD,cpsE,cpsF,cpsG,cpsH,cpsI,cpsJ,cpsK,ebpA,ebpB,ebpC,efaA,fss1,fss2,prgB/asc10,srtC

NXC 42	aac(6')-Ii,msr(C)	acm
NXC 43	aac(6')-Ii,msr(C)	acm
NXC 44	aac(6')-Ii,msr(C),str,tet(L),tet(M)	acm
NXC 45	aac(6')-Ii,msr(C)	acm
NXC 46	aac(6')-Ii,lnu(G),msr(C)	acm,ecbA
NXC 47	ant(6)-Ia,erm(B),lsa(A),tet(S)	bopD,cpsA,cpsB,ebpA,ebpB,ebpC,efaA,fsrC,fss1,fss3,gelE,sprE,srtC
NXC 48	lsa(A)	EF3023,ace,bopD,cpsA,cpsB,ebpA,ebpB,ebpC,efaA,fsrA,fsrB,fsrC,fss1,gelE,sprE,srtC
NXC 49	lsa(A)	EF3023,ace,bopD,cpsA,cpsB,ebpA,ebpB,ebpC,efaA,fsrA,fsrB,fsrC,fss1,gelE,sprE,srtC
NXC 50	tet(M)	-

NXC 51	aac(6')-Iid	-
NXC 52	aac(6')-Iid	-
NXC 53	aac(6')-Iid	fss3
NXC 54	aac(6')-aph(2''),aph(3')-III,dfrG,erm(B),lnu(B),lsa(A),lsa(E),tet(L),tet(M)	EF3023,bopD,cpsA,cpsB,ebpA,ebpB,ebpC,efaA,fss1,srtC
NXC 55	dfrG,lsa(A),tet(L),tet(M)	EF3023,bopD,cpsA,cpsB,ebpA,ebpB,ebpC,efaA,fss1,srtC
NXC 56	aac(6')-aph(2''),dfrG,erm(B),lsa(A),tet(L),tet(M)	EF3023,bopD,cpsA,cpsB,ebpA,ebpB,ebpC,efaA,fss1,srtC
NXC 57	aac(6')-Iid	-

NXC 58	aac(6')-aph(2''),aph(3')-III,dfrG,erm(B),lnu(B),lsa(A),lsa(E),tet(L),tet(M)	EF3023,bopD,cpsA,cpsB,ebpA,ebpB,ebpC,efaA,fss1,srtC
NXC 59	aac(6')-aph(2''),aph(3')-III,dfrG,erm(B),lnu(B),lsa(A),lsa(E),tet(L),tet(M)	EF3023,bopD,cpsA,cpsB,ebpA,ebpB,ebpC,efaA,fss1,srtC
NXC 5	aac(6')-Ii,msr(C)	acm
NXC 60	aac(6')-Ii,msr(C)	acm
NXC 61	aac(6')-Ii,msr(C)	ecbA
NXC 62	aac(6')-Iid	-
NXC 63	aac(6')-Iid	-

NXC 64	aac(6')-aph(2''),aph(3')-III,cat,dfrG,erm(B),lnu(B),lsa(A),lsa(E),str,tet(M)	EF0485,EF3023,bopD,cpsA,cpsB,cpsC,cpsD,cpsE,cpsF,cpsG,cpsH,cpsI,cpsJ,cpsK,cylA,cylB,cylI,cylL,cylM,cylR1,cylR2,cylS,ebpA,ebpB,ebpC,efaA,fss1,srtC
NXC 65	aac(6')-Iid,erm(B),tet(L),tet(M)	-
NXC 66	aac(6')-aph(2''),ant(9)-Ia,aph(3')-III,cat,erm(A),erm(B),fexA,lnu(B),lsa(A),lsa(E),optrA,tet(M)	EF0485,EF3023,bopD,cpsA,cpsB,cpsC,cpsD,cpsE,cpsF,cpsG,cpsH,cpsI,cpsJ,cpsK,cylA,cylB,cylL,cylM,cylR1,cylR2,cylS,ebpA,ebpB,ebpC,efaA,fss1,fss2,prgB/asc10,srtC
NXC 67	aac(6')-aph(2''),ant(9)-Ia,aph(3')-III,cat,erm(A),erm(B),fexA,lnu(B),lsa(A),lsa(E),optrA,tet(M)	EF0485,EF3023,asa1,bopD,cpsA,cpsB,cpsC,cpsD,cpsE,cpsF,cpsG,cpsH,cpsI,cpsJ,cpsK,cylA,cylB,cylI,cylL,cylM,cylR1,cylR2,cylS,ebpA,ebpB,ebpC,efaA,fss1,fss2,prgB/asc10,srtC
NXC 71	lsa(A)	EF0818,EF3023,ace,bopD,cpsA,cpsB,ebpA,ebpB,ebpC,efaA,fsrA,fsrB,fsrC,fss1,gelE,sprE,srtC
NXC 72	ant(6)-Ia,aph(3')-III,cat,dfrG,erm(B),fexA,lsa(A),optrA,str,tet(L),tet(M)	EF0485,EF0818,EF3023,bopD,cpsA,cpsB,cpsC,cpsD,cpsE,cpsF,cpsG,cpsH,cpsI,cpsJ,cpsK,cylI,ebpA,ebpB,ebpC,efaA,fsrA,fsrB,fsrC,fss1,fss2,gelE,sprE,srtC

NXC 73	lsa(A)	EF0818,EF3023,ace,bopD,cpsA,cpsB,ebpA,ebpB,ebpC,efaA,fsrA,fsrB,fsrC,fss1,fss2,gelE,sprE,srtC
NXC 74	aac(6')-Iid,tet(L),tet(M)	-
NXC 75	lsa(A)	EF0818,EF3023,asa1,bopD,cpsA,cpsB,cylI,ebpA,ebpB,ebpC,efaA,fsrA,fsrB,fsrC,fss1,gelE,sprE,srtC
NXC 76	aac(6')-Ii,msr(C)	acm
NXC 77	aac(6')-Iid	fss3
NXC 78	lsa(A)	EF0818,EF3023,bopD,cpsA,cpsB,ebpA,ebpB,ebpC,efaA,fsrA,fsrB,fsrC,fss1,fss2,gelE,sprE,srtC
NXC 7	erm(B),lsa(A),tet(M)	EF0485,EF3023,bopD,cpsA,cpsB,cpsC,cpsD,cpsE,cpsF,cpsG,cpsH,cpsI,cpsJ,cpsK,cylA,cylB,cylI,cylL,cylM,cylR1,cylR2,cylS,ebpA,ebpB,ebpC,efaA,fss1,fss2,srtC
NXC 81	aac(6')-aph(2''),ant(6)-Ia,ant(9)-Ia,aph(3')-III,cat,dfrG,erm(A),erm(B),fexA,lsa(A),optrA,tet(L),tet(M)	EF0485,EF0818,ace,bopD,cpsA,cpsB,cpsC,cpsD,cpsE,cpsG,cpsH,cpsI,cpsJ,cpsK,cylI,ebpA,ebpB,ebpC,efaA,fsrA,fsrB,fsrC,fss1,fss2,gelE,prgB/asc10,sprE,srtC

NXC 84	aac(6')-Iid,ant(9)-Ia,dfrG,erm(A),erm(B),tet(L),tet(M)	-
NXC 85	aac(6')-aph(2''),aph(3')-III,cat,dfrG,erm(B),lnu(B),lsa(A),lsa(E),tet(L),tet(M)	EF3023,bopD,cpsA,cpsB,ebpA,ebpB,ebpC,efaA,fss1,srtC
NXC 86	ant(9)-Ia,cat,dfrG,erm(A),fexA,lsa(A),optrA,tet(L),tet(M)	EF0818,EF3023,bopD,cpsA,cpsB,ebpA,ebpB,ebpC,ecbA,efaA,fsrA,fsrB,fsrC,fss1,gelE,sprE,srtC
NXC 87	aac(6')-Ii,msr(C)	acm
NXC 88	aac(6')-aph(2''),aph(3')-III,cat,dfrG,erm(B),lnu(B),lsa(A),lsa(E),tet(L),tet(M)	EF3023,bopD,cpsA,cpsB,ebpA,ebpB,ebpC,ecbA,efaA,fss1,srtC
NXC 89	ant(6)-Ia,aph(3')-III,cat,dfrG,erm(A),erm(B),fexA,lsa(A),optrA,tet(L),tet(M)	EF0818,EF3023,bopD,cpsA,cpsB,cpsC,cpsD,cpsE,cpsG,cpsH,cpsI,cpsJ,cpsK,ebpA,ebpB,ebpC,efaA,fsrA,fsrB,fsrC,fss1,fss2,gelE,prgB/asc10,sprE,srtC

NXC 8	aac(6')-Ii,msr(C)	acm
NXC 90	aac(6')-aph(2''),ant(9)-Ia,aph(3')-III,cat,dfrG,erm(A),erm(B),fexA,lsa(A),optrA,str,tet(L),tet(M)	EF3023,bopD,cpsA,cpsB,cpsC,cpsD,cpsE,cpsF,cpsG,cpsH,cpsI,cpsJ,cpsK,ebpA,ebpB,ebpC,efaA,fss1,srtC
NXC 91	ant(6)-Ia,aph(3')-III,cat,dfrG,erm(A),fexA,lsa(A),optrA,tet(L),tet(M)	EF0818,EF3023,bopD,cpsA,cpsB,cpsC,cpsD,cpsE,cpsG,cpsH,cpsI,cpsJ,cpsK,ebpA,ebpB,ebpC,efaA,fsrA,fsrB,fsrC,fss1,fss2,gelE,prgB/asc10,sprE,srtC
NXC 92	aac(6')-aph(2''),aph(3')-III,lsa(A),tet(L),tet(M)	EF0818,EF3023,asa1,bopD,cpsA,cpsB,cylA,cylB,cylI,cylL,cylM,cylR1,cylR2,cylS,ebpA,ebpB,ebpC,efaA,fsrA,fsrB,fsrC,fss1,gelE,sprE,srtC
NXC 95	cat,dfrG,erm(A),erm(B),fexA,lsa(A),optrA,str,tet(L),tet(M)	EF3023,bopD,cpsA,cpsB,cpsC,cpsD,cpsE,cpsF,cpsG,cpsH,cpsI,cpsJ,cpsK,ebpA,ebpB,ebpC,efaA,fss1,srtC
NXC 99	aac(6')-aph(2''),aph(3')-III,cat,dfrG,erm(A),erm(B),fexA,lnu(B),lsa(A),lsa(E),optrA,str,tet(M)	EF0485,EF3023,bopD,cpsA,cpsB,cpsC,cpsD,cpsE,cpsF,cpsG,cpsH,cpsI,cpsJ,cpsK,cylA,cylB,cylI,cylL,cylM,cylR1,cylR2,cylS,ebpA,ebpB,ebpC,efaA,fss1,fss2,srtC

NXC 9	aac(6')-aph(2''),ant(6)-Ia,aph(3')-III,dfrG,lnu(B),lsa(A),lsa(E),str,tet(L),tet(M)	EF0485,EF0818,EF3023,bopD,cpsA,cpsB,cylI,ebpA,ebpB,ebpC,efaA,fsrA,fsrB,fsrC,fss1,gelE,prgB/asc10,sprE,srtC
NXGC33	ant(6)-Ia,aph(3')-III,erm(B),lnu(G),lsa(A),tet(L),tet(M)	EF3023,bopD,cpsA,cpsB,cpsC,cpsD,cpsE,cpsF,cpsG,cpsH,cpsI,cpsJ,cpsK,ebpA,ebpB,ebpC,efaA,fsrA,fsrB,fsrC,fss1,gelE,prgB/asc10,sprE,srtC
NXGC34	lsa(A)	EF0818,ace,bopD,cpsA,cpsB,cpsC,cpsD,cpsE,cpsG,cpsH,cpsI,cpsJ,cpsK,ebpA,ebpB,ebpC,efaA,fsrA,fsrB,fsrC,fss1,gelE,sprE,srtC
NXGC35	dfrG,erm(B),lsa(A),tet(L),tet(M)	EF0149,EF0485,EF0818,bopD,cpsA,cpsB,cpsC,cpsD,cpsE,cpsG,cpsH,cpsI,cpsJ,cpsK,cylI,ebpA,ebpB,ebpC,efaA,fsrA,fsrB,fsrC,fss1,gelE,sprE,srtC
NXGC36	ant(6)-Ia,aph(3')-III,erm(B),lnu(G),lsa(A),tet(L),tet(M)	EF3023,bopD,cpsA,cpsB,cpsC,cpsD,cpsE,cpsF,cpsG,cpsH,cpsI,cpsJ,cpsK,ebpA,ebpB,ebpC,efaA,fsrA,fsrB,fsrC,fss1,gelE,sprE,srtC
NXGC37	aac(6')-Ii,ant(6)-Ia,erm(B),lnu(B),lsa(E),msr(C),tet(L),tet(M)	acm

NXGC38	ant(9)-Ia,aph(3')-III,cat,dfrG,erm(A),erm(B),fexA,lsa(A),optrA,tet(L),tet(M)	EF3023,bopD,cpsA,cpsB,cpsC,cpsD,cpsE,cpsF,cpsG,cpsH,cpsI,cpsJ,cpsK,ebpA,ebpB,ebpC,efaA,fsrA,fsrB,fsrC,fss1,gelE,sprE,srtC
NXGC39	cat,erm(B),lsa(A),tet(M)	EF3023,bopD,cpsA,cpsB,cpsC,cpsD,cpsE,cpsF,cpsG,cpsH,cpsI,cpsJ,cpsK,cylA,cylB,cylI,cylL,cylM,cylR1,cylR2,cylS,ebpA,ebpB,ebpC,efaA,fsrA,fsrB,fsrC,fss1,fss2,gelE,sprE,srtC
NXGC40	aac(6')-Ii,erm(B),lnu(B),lsa(E),msr(C),tet(L)	acm
